# Association of Glomerular Filtration Rate Decline With Clinical Outcomes in a Population With Type 2 Diabetes

**DOI:** 10.1177/20543581241255781

**Published:** 2024-06-10

**Authors:** Scott W. Klarenbach, David Collister, Natasha Wiebe, Aminu Bello, Stephanie Thompson, Neesh Pannu

**Affiliations:** 1 Department of Medicine, University of Alberta, Edmonton, Canada

**Keywords:** type 2 diabetes, eGFR slope, kidney failure, cardiovascular events, hospitalization, logistic models

## Abstract

**Background:**

While historical rate of decline in kidney function is informally used by clinicians to estimate risk of future adverse clinical outcomes especially kidney failure, in people with type 2 diabetes the epidemiology and independent association of historical eGFR slope on risk is not well described.

**Objective:**

Determine the association of eGFR slope and risk of clinically important outcomes.

**Design Setting and Patients:**

Observational population-based cohort with type 2 diabetes in Alberta.

**Measurement and Methods:**

An Alberta population-based cohort with type 2 diabetes was assembled, characterized, and observed over 1 year (2018) for clinical outcomes of ESKD, first myocardial infarction, first stroke, heart failure, and disease-specific and all-cause hospitalization and mortality. Kidney function was defined using KDIGO criteria using the most recent eGFR and albuminuria measured in the preceding 18 months; annual eGFR slope utilized measurements in the 3 years prior and was parameterized using three methods (percentiles, and linear term with and without missingness indicator). Demographics, laboratory results, medications, and comorbid conditions using validated definitions were described. In addition to descriptive analysis, odds ratios from fully adjusted logistic models regressing outcomes on eGFR slope are reported; the marginal risk of clinical outcomes was also determined.

**Results:**

Among 336 376 participants with type 2 diabetes, the median annual eGFR slope was −0.41 mL/min/1.73 m^2^ (IQR −1.67, 0.62). In fully adjusted models, eGFR slope was independently associated with many adverse clinical outcomes; among those with ≤10th percentile of slope (median −4.71 mL/min/1.73 m^2^) the OR of kidney failure was 2.22 (95% CI 1.75, 2.82), new stroke 1.23 (1.08, 1.40), heart failure 1.42 (1.27, 1.59), MI 0.98 (0.77, 1.23) all-cause hospitalization 1.31 (1.26, 1.36) and all-cause mortality 1.56 (1.44, 1.68). For every −1 mL/min/1.73 m^2^ in eGFR slope, the OR of outcomes ranged from 1.01 (0.98, 1.05 for new MI) to 1.09 (1.08, 1.10 for all-cause mortality); findings were significant for 10 of the 13 outcomes considered.

**Limitations:**

Causality cannot be established with this study design.

**Conclusions:**

These findings support consideration of the rate of eGFR decline in risk stratification and may inform clinicians and policymakers to optimize treatment and inform health care system planning.

## Abrege

**Contexte:**

Bien que les antécédents de déclin de la fonction rénale soient utilisés de manière informelle par les cliniciens pour estimer le risque d’issues cliniques défavorables — particulièrement l’insuffisance rénale terminale (IRT) — chez les diabétiques de type 2, l’épidémiologie de la pente du DFGe et son association indépendante sur ce risque demeurent mal décrites.

**Objectif:**

Examiner l’association entre la pente du DFGe et le risque de résultats d’importance clinique.

**Sujets et conception de l’étude:**

Étude de cohorte observationnelle basée sur une population d’Albertains atteints de diabète de type 2.

**Méthodologie et mesures:**

Nous avons constitué, caractérisé et observé une cohorte d’Albertains atteints de diabète de type 2 sur une période d’un an (2018) pour les résultats cliniques suivants: IRT, premier infarctus du myocarde (IM), premier AVC, insuffisance cardiaque, ainsi que les hospitalisations et la mortalité liées à la maladie et à toutes causes confondues. La fonction rénale a été définie selon les critères KDIGO à partir des plus récentes valeurs de DFGe et d’albuminurie mesurées dans les 18 mois précédents. La pente annuelle du DFGe a été calculée à partir des mesures effectuées au cours des trois années précédentes et paramétrée selon trois méthodes (percentiles, termes linéaires avec et sans indications de données manquantes). Les données démographiques, les résultats de laboratoire, les médicaments et les comorbidités ont été décrits selon les définitions validées. En plus de l’analyze descriptive, des rapports de cotes (RC) pour les résultats liés au déclin du DFGe ont été établis à l’aide de modèles de régression logistique entièrement ajustés; le risque marginal des résultats cliniques d’intérêt a également été déterminé.

**Résultats:**

Parmi les 336 376 diabétiques de type 2 participants, la pente annuelle médiane du DFGe s’établissait à −0,41 ml/min/1,73 m^2^ (ÉIQ: −1,67 à 0,62). Dans les modèles ajustés, la pente du DFGe a été associée de façon indépendante à plusieurs issues cliniques défavorables. Parmi ceux qui présentaient une pente du DFGe ≤10^e^ percentile (médiane: −4,71 ml/min/1,73 m^2^), le RC était de 2,22 (IC 95 %: 1,75 à 2,82) pour l’insuffisance rénale; de 1,23 (1,08 à 1,40) pour les nouveaux AVC; de 1,42 (1,27 à 1,59) pour l’insuffisance cardiaque; de 0,98 (0,77 à 1,23) pour les nouveaux IM; de 1,31 (1,26 à 1,36) pour les hospitalisations toutes causes confondues et de 1,56 (1,44 à 1,68) pour la mortalité toutes causes confondues. Pour chaque tranche de - 1 ml/min/1,73 m^2^ de la pente du DFGe, le RC des résultats cliniques variait de 1,01 (0,98 à 1,05) pour les nouveaux IM à 1,09 (1,08 à 1,10) pour la mortalité toutes causes confondues; les résultats étaient significatifs pour 10 des 13 résultats examinés.

**Limites:**

La causalité ne peut pas être établie avec ce plan d’étude.

**Conclusion:**

Ces résultats plaident en faveur de la prise en compte du taux de déclin du DFGe dans la stratification du risque. Ils peuvent également aider les cliniciens et les décideurs à optimiser le traitement et à planifier les systèmes de soins de santé.

## Introduction

The incidence and prevalence of type 2 diabetes mellitus (T2DM) is increasing worldwide and in North America, which impacts the epidemiology of chronic kidney disease (CKD);^1^ approximately half of people with T2DM have concomitant CKD.^2^ Persons with both T2DM and CKD have a high risk of adverse events, including kidney failure, cardiovascular events, mortality, and healthcare resource use.^3^ Assessing the risk of future adverse clinical events may inform the appropriate use of preventative treatment and processes of care and guide health care resource planning. People with T2DM and CKD are commonly classified by KDIGO risk categories, incorporating GFR as well as the severity of albuminuria. However, other factors may also inform risk, including historical rate of loss of kidney function.

There is known variability in the rate of progression of kidney disease in persons with T2DM; however, the distribution of the rate of progression in a population-based cohort and the relationship to the KDIGO risk category is not well described. While the rate of decline in GFR is frequently used informally by clinicians as an indicator of future risk of kidney failure, the association between rate of progression of kidney disease and other adverse clinical outcomes including cardiovascular events, hospitalization, and mortality is not well described.

Understanding the distribution of the rate of progression of CKD among patients with T2DM, how the severity of progression at baseline associates with KDIGO risk categories, treatment patterns, kidney and cardiovascular outcomes, and healthcare resource use would be useful to clinicians and policymakers to identify opportunities to improve care and optimize outcomes in this growing patient population. We sought to contribute to previously published research to consider a contemporary population-based cohort with T2DM to examine a wide range of clinically important outcomes.^4-7^

## Methods

This retrospective cohort study is reported according to the STROBE guidelines^8^ and was conducted according to an *a* priori study protocol. The institutional review board at the University of Alberta (Pro00106318) approved this study and waived the requirement for participants to provide consent.

### Data Source for the Cohort

We used the Alberta Kidney Disease Network database, which incorporates registry, physician claims, hospitalizations, ambulatory care utilization, and pharmaceutical information network (outpatient prescription dispensation) files from all adults registered with Alberta Health (AH; the provincial health ministry) and links them with data from provincial clinical laboratories and provincial kidney programs. This database has been widely used^3,9,10^ because of its population-based coverage of a geographically defined area, including demographic characteristics, health services utilization, and clinical outcomes. Additional information on the database is available elsewhere, including the validation of selected data elements and the standardization and calibration of serum creatinine assays.^11^ All Alberta residents are eligible for insurance coverage by AH, and >99% participate in coverage. The database was used to assemble cohorts of adults (≥18 years) with T2DM who resided in Alberta, Canada, on December 31, 2017 (index). Participants were excluded if they had type 1 diabetes (ICD-9-CM 250.x1, 250.x3, or ICD-10-CA E10) prior to T2DM or if they had initiated renal replacement therapy prior to index (chronic dialysis or in receipt of a kidney transplant). All participants were followed until December 31, 2018, death or out-migration, whichever occurred sooner (Supplement Figure 1).

### Type 2 Diabetes, Comorbidities, and Other Exposures

T2DM (ICD-9-CM 250.x0, 250.x2 or ICD-10-CA E11, E12, E14) and baseline comorbidities were defined using a previously published framework using validated algorithms as applied to Canadian physician claims, hospitalizations, and ambulatory care data, each of which had positive predictive values ≥70% as compared to a gold standard measure, such as chart review.^12^ Comorbidities included atrial fibrillation,^13,14^ chronic heart failure,^14,15^ myocardial infarction,^15^ peripheral artery disease,^16^ hypertension,^12^ and stroke.^17^ Diabetic retinopathy was included although the algorithm (1 hospitalization or 1 ACCS or 2 claims in 2 years with one of the following codes: ICD-9-CM 250.5, 362.0, 362.81, 379.23 or ICD-10-CA E10.3, E11.3, E14.3, H35.6, H36.0, H43.1, H45.0) has not been validated.^18^ Myocardial infarction was included in the definition of coronary artery disease along with percutaneous coronary intervention (ICD-9 procedures codes: 36.01, 36.02, 36.05, 36.06, and CCI 1.IJ.50, 1.IJ.57.GQ, 1.IL.35) and coronary artery bypass grafting (ICD-9 procedures codes: 36.1, 36.2, and CCI 1.IJ.76). Cardiovascular disease was defined as atrial fibrillation, chronic heart failure, coronary artery disease, peripheral artery disease, and stroke. Each participant was classified with respect to the presence or absence of these seven chronic conditions at baseline (lookback extended as far as April 1994 when records were available).^19^

The categorization of CKD was based on the estimated GFR and the presence or absence of albuminuria according to the KDIGO guidelines.^20^ Category of CKD was based on the participant’s most recent outpatient eGFR (if available) within 18 months of the index date: ≥90 mL/min/1.73 m^2^ (stage 1), 60-<90 mL/min/1.73 m^2^ (stage 2), 45-<60 mL/min/1.73 m^2^ (stage 3a), 30-<45 mL/min/1.73 m^2^ (stage 3b), 15-<30 mL/min/1.73 m^2^ (stage 4), and <15 mL/min/1.73 m^2^ (stage 5). eGFR was calculated using the CKD-Epidemiology Collaboration (EPI) equation without race adjustment.^21^ Albuminuria (if available) was captured using the participant’s most recent outpatient measurement within 18 months of the index date using either ACR, the protein: creatinine ratio (PCR), or dipstick. The PCR assessment was used when ACR was not available, and dipstick results were used when PCR was not available. Measurements were categorized as follows: missing, none/mild (ACR <3 mg/mmol, PCR <15 mg/mmol, dipstick negative/trace), moderate (ACR 3-30 mg/mmol, PCR 15-50 mg/mmol, dipstick 1+), severe (ACR 31-220 mg/mmol, PCR 51-350 mg/mmol, dipstick 2+ and 3+), and nephrotic (ACR >220 mg/mmol, PCR >350 mg/mmol, dipstick ≥4+). The KDIGO guidelines combine eGFR and albuminuria into four risk categories: very-high risk (CKD stage 4 or 5, CKD stage 3b plus moderate albuminuria, CKD stage 3a plus severe albuminuria), high risk (CKD stage 3b, CKD stage 3a plus moderate albuminuria, severe albuminuria), moderately increased risk (CKD stage 3a, moderate albuminuria), and low risk (CKD stage 1 or 2 plus none/mild albuminuria). A time frame of 18 months was used for eGFR and ACR determinations as they are infrequently captured in some patients; using a longer time frame increases the proportion of subjects with available data and may lead to a more accurate determination of baseline eGFR while still representing characteristics at baseline.

The rate of decline in eGFR before the index date was based on all available outpatient eGFR measurements over the previous 3 years. A 3-year period was used to assess change in eGFR (per year), allowing a contemporaneous assessment while maximizing data from measurements that may be infrequent. A minimum of three values were required. The median was 5, with an interquartile range of 4 to 8. The eGFR data was regressed on participants and time using mixed effects models. Both participant and participant time were modeled as random effects. The latter was annualized and formed the estimate for each participant’s estimate of baseline eGFR slope. Glycated hemoglobin (A1c) was also included as an exposure.

Prescriptions included sodium-glucose cotransporter-2 (SGLT2) inhibitors, angiotensin-converting enzyme (ACE) inhibitors, angiotensin II receptor blockers (ARB), and statins. Each participant was classified with baseline use if there was a new prescription within 120 days prior to baseline. As in our prior work, we used administrative data to identify age, biological sex, and rural residence location.^21^

### Clinical Outcomes

A 1-year time period after the assessment of baseline characteristics was used for outcome ascertainment, from January 1, 2018, to December 31, 2018. Outcomes included incidence of end-stage kidney disease, first myocardial infarction, first stroke, and new diagnosis of heart failure in participants without prior occurrences of these events. End-stage kidney disease was defined as the initiation of chronic dialysis, receipt of kidney transplant, or at least two measures of eGFR <15 mL/min/1.73 m^2^ 30 days or more apart. Other outcomes were hospitalizations (all-cause, kidney, and cardiovascular-specific, defined as myocardial infarction, stroke, or heart failure) and mortality (all-cause, renal, and cardiovascular-specific).

### Statistical Analyses

We did analyses with Stata MP 17·0 (www.stata.com) and reported baseline descriptive statistics as percentages, or medians and interquartile ranges, as appropriate. A spine plot is reported to explore the association of the annual eGFR slope (categorized as ≥50th, 26th-50th, 11th-25th, and ≤10th percentiles) with KDIGO risk. The participants in the ≤10th percentile group had the greatest declines in eGFR. Clinical outcomes were regressed on the annual eGFR slope, exploring three different parametrizations of the eGFR slope. Model 1 parameterization used percentiles—categorized as ≥50th, 26th-50th, 11th-25th, ≤10th percentiles, and missing. Model 2 parameterization used a linear term for slope and an indicator term for missingness. Slopes that were positive (all in the ≥50th percentiles group) were assigned a value of 0. Model 3 also adjusted for a linear term, but positive slopes and missing slopes were assigned a value of 0. All of the models were further adjusted for baseline eGFR (≥90, 60-<90, 45-<60, 30-<45, 15-<30, <15 mL/min/1.73 m^2^, and missing), albuminuria (none/mild, moderate, severe, missing), A1c (<7%, 7-8%, >8-9%, >9%, and missing), age, biological sex, rural status, comorbidities (atrial fibrillation, heart failure, coronary artery disease, peripheral artery disease, stroke, retinopathy) and prescriptions filled (SGLT2 inhibitors, ACE inhibitors or ARBs, statins). Odds ratios with 95% confidence intervals are reported. The threshold *p* for statistical significance was set at 0.05.

In sensitivity analyses using Model 2s parameterization, we considered (1) participants with and without ACEi, ARB, or SGLT2i prescriptions and (2) did an analysis using only the covariates from the Kidney Failure Risk Equation^22^: age, sex, baseline eGFR, and albuminuria.

## Results

### Characteristics of Participants

**Figure 1.**
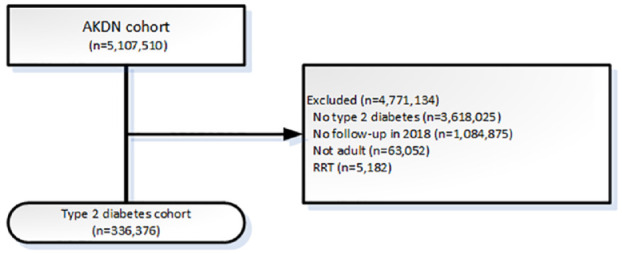
Participant flow. AKDN = Alberta Kidney Disease Network; RRT = renal replacement therapy (receipt of kidney transplant or chronic dialysis).

**Figure 2.**
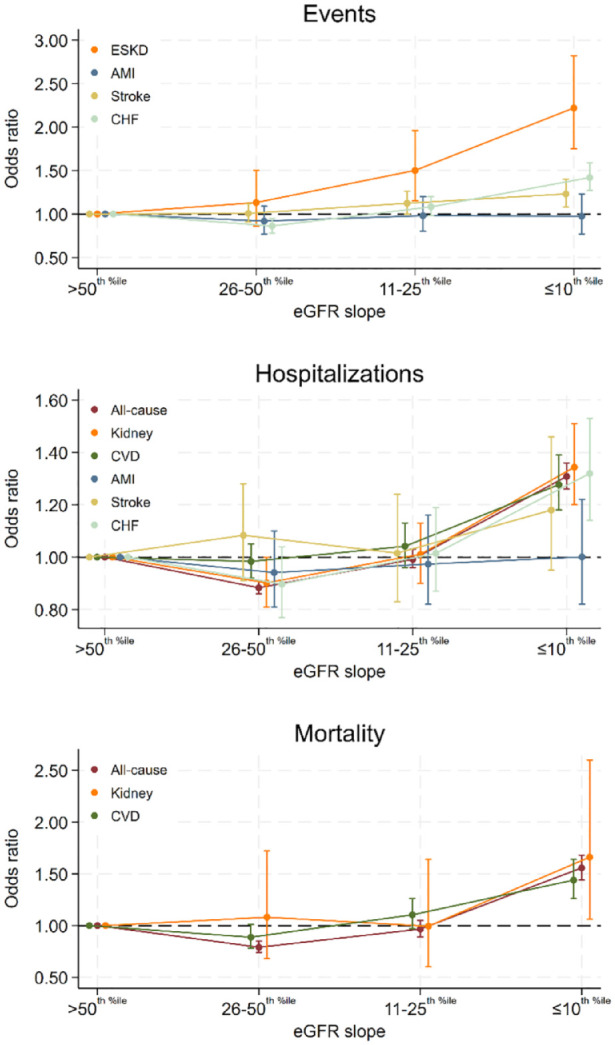
Adjusted odds ratios of clinical outcomes by eGFR slope. ACE = angiotensin-converting enzyme; ARB = angiotensin II receptor blockers; eGFR = estimated glomerular filtration rate; ESKD = end-stage kidney disease; HbA1c = glycated hemoglobin; MI = myocardial infarction; SGLT2 = sodium-glucose cotransporter-2; TIA = transient ischemic attack. Odds ratios with 95% confidence intervals are presented for various model parametrizations of the eGFR slope. The model parametrizes eGFR slope into 5 percentile bins: ≥50th percentile (range −0.41 to 26.22 mL/min/1.73 m^2^; referent), 26th-50th percentiles (range −1.6 to −0.41 mL/min/1.73 m)^2^, 11th-25th percentiles (range −3.34 to −1.67 mL/min/1.73 m^2^), ≤10th percentile (range −48.04 to −3.34 mL/min/1.73 m)^2^, and missing slope data (not shown). The model adjusts for baseline eGFR (≥90, 60-<90, 45-<60, 30-<45, 15-<30, <15 mL/min/1.73 m^2^, and missing), albuminuria (none/mild, moderate, severe, missing), glycated hemoglobin (<7%, 7-8%, >8-9%, >9%, and missing), age, biological sex, rural status, comorbidities (atrial fibrillation, heart failure, coronary artery disease, peripheral artery disease, stroke, and retinopathy), and prescriptions filled (SGLT2 inhibitors, ACE inhibitors or ARBs, and statins).

Participant flow is shown in Figure 1. There were 336 376 adults with T2DM residing in Alberta in 2018 without prior RRT or type 1 diabetes; out-migration occurred in 0.3% of participants. The median age was 63 years (IQR 53, 73), and 46.7% were women (Table 1). Most (86.6%) resided in towns or cities. Diabetes vintage was a median of 8 years (IQR 4, 13); median A1c was 6.8% (IQR 6.1, 7.8). Participants, on average, had 1 comorbidity: 71.3% had hypertension, 27.1% had cardiovascular disease, and 11.4% had diabetic retinopathy. Baseline prescription use was 55.4% for ACE inhibitors or ARBs, 52.0% for statins, and 11.3% for SGLT2 inhibitors. Over the 1-year follow-up, 2.6% experienced a first event, 12.8% were hospitalized, and 2.3% died (Figure 2; Supplement Table 1).

**Table 1.** Demographics and Clinical Characteristics by eGFR Slope.

Characteristics	All	eGFR slope, mL/min/1.73 m^2^/year				
		>50th %ile	26th-50th %ile	11th-25th %il^e^	≤10th %ile	Missing
N	336 376	109 741 (32.6)	54 871 (16.3)	32 922 (9.8)	21 948 (6.5)	116 894 (34.8)
eGFR slope						
Median	–0.41	0.62	–0.95	–2.31	–4.71	
Range	(−109.06, 26.22)	(−0.41, 26.22)	(−1.67,−0.41)	(−3.34,−1.67)	(−48.04,−3.34)	–
Age, years						
18–39	27 423 (8.2)	5624 (5.1)	2481 (4.5)	1478 (4.5)	982 (4.5)	16 858 (14.4)
40–64	159 395 (47.4)	49 926 (45.5)	24 287 (44.3)	13 515 (41.1)	8684 (39.6)	62 983 (53.9)
≥65	149 558 (44.5)	54 191 (49.4)	28 103 (51.2)	17 929 (54.5)	12 282 (56.0)	37 053 (31.7)
Women	157 215 (46.7)	50 974 (46.4)	25 279 (46.1)	15 404 (46.8)	10 579 (48.2)	54 979 (47.0)
Rural residence	45 193 (13.4)	14 860 (13.5)	6995 (12.7)	4608 (14.0)	3576 (16.3)	15 154 (13.0)
Duration of diabetes, years	8 [4, 13]	8 [4, 13]	8 [4, 14]	9 [4, 15]	10 [5, 17]	7 [3, 12]
HbA1c, %						
<7	149 597 (44.5)	58 885 (53.7)	28 474 (51.9)	16 344 (49.6)	10 104 (46.0)	35 790 (30.6)
7-8	61 619 (18.3)	23 312 (21.2)	12 837 (23.4)	7727 (23.5)	5220 (23.8)	12 523 (10.7)
>8-9	25 281 (7.5)	8914 (8.1)	4990 (9.1)	3307 (10.0)	2412 (11.0)	5658 (4.8)
>9	28 816 (8.6)	8926 (8.1)	4579 (8.3)	3203 (9.7)	2692 (12.3)	9416 (8.1)
Missing	71 063 (21.1)	9704 (8.8)	3991 (7.3)	2341 (7.1)	1520 (6.9)	53 507 (45.8)
eGFR, mL/min/1.73 m^2^						
≥90	101 567 (30.2)	44 909 (40.9)	18 842 (34.3)	5815 (17.7)	1411 (6.4)	30 590 (26.2)
60-<90	119 820 (35.6)	45 693 (41.6)	23 551 (42.9)	16 877 (51.3)	9418 (42.9)	24 281 (20.8)
45-<60	30 934 (9.2)	10 178 (9.3)	6505 (11.9)	5506 (16.7)	5332 (24.3)	3413 (2.9)
30-<45	15 404 (4.6)	4498 (4.1)	3427 (6.2)	2967 (9.0)	3517 (16.0)	995 (0.9)
15-<30	5480 (1.6)	1188 (1.1)	1226 (2.2)	1178 (3.6)	1719 (7.8)	169 (0.1)
<15	972 (0.3)	80 (0.1)	183 (0.3)	255 (0.8)	438 (2.0)	16 (0.0)
Missing	62 199 (18.5)	3195 (2.9)	1137 (2.1)	324 (1.0)	113 (0.5)	57 430 (49.1)
Albuminuria						
None/mild	178 987 (53.2)	69 998 (63.8)	34 896 (63.6)	19 570 (59.4)	10 941 (49.8)	43 582 (37.3)
Moderate	43 654 (13.0)	16 626 (15.2)	8965 (16.3)	5913 (18.0)	4485 (20.4)	7665 (6.6)
Severe	15 515 (4.6)	4686 (4.3)	2877 (5.2)	2587 (7.9)	3354 (15.3)	2011 (1.7)
Missing	98 220 (29.2)	18 431 (16.8)	8133 (14.8)	4852 (14.7)	3168 (14.4)	63 636 (54.4)
Comorbidities	1 [0, 2]	1 [1, 2]	1 [1, 2]	1 [1, 2]	1 [1, 3]	1 [0, 1]
Cardiovascular disease	91 097 (27.1)	33 251 (30.3)	16 381 (29.9)	11 493 (34.9)	9967 (45.4)	20 005 (17.1)
Atrial fibrillation	25 723 (7.7)	9529 (8.7)	4702 (8.6)	3473 (10.5)	3377 (15.4)	4642 (4.0)
Chronic heart failure	31 919 (9.5)	11 085 (10.1)	5517 (10.1)	4470 (13.6)	5207 (23.7)	5640 (4.8)
Coronary artery disease	31 917 (9.5)	11 590 (10.6)	5860 (10.7)	4218 (12.8)	3645 (16.6)	6604 (5.6)
Myocardial infarction	19 144 (5.7)	6812 (6.2)	3374 (6.1)	2446 (7.4)	2156 (9.8)	4356 (3.7)
Peripheral artery disease	9705 (2.9)	3567 (3.3)	1758 (3.2)	1338 (4.1)	1284 (5.9)	1758 (1.5)
Stroke/TIA	40 121 (11.9)	14 915 (13.6)	7103 (12.9)	4844 (14.7)	4088 (18.6)	9171 (7.8)
Diabetic retinopathy	46 784 (13.9)	15 960 (14.5)	8629 (15.7)	6170 (18.7)	5130 (23.4)	10 895 (9.3)
Hypertension	239 947 (71.3)	85 813 (78.2)	43 645 (79.5)	27 185 (82.6)	19 217 (87.6)	64 087 (54.8)
Prescriptions filled						
SGLT2 inhibitor	38 100 (11.3)	14 217 (13.0)	7775 (14.2)	4926 (15.0)	3394 (15.5)	7788 (6.7)
ACE inhibitor or ARB	186 340 (55.4)	69 033 (62.9)	36 319 (66.2)	22 926 (69.6)	15 964 (72.7)	42 098 (36.0)
Statins	174 776 (52.0)	66 916 (61.0)	34 734 (63.3)	21 329 (64.8)	14 500 (66.1)	37 297 (31.9)

*Note.* ACE = angiotensin-converting enzyme; ARB = angiotensin II receptor blockers; eGFR = estimated glomerular filtration rate; HbA1c = glycated hemoglobin; KDIGO = Kidney Disease Improving Global Outcomes; SGLT2 = sodium-glucose cotransporter-2; TIA = transient ischemic attack. n (%) or median [interquartile range].

**Figure 3.**
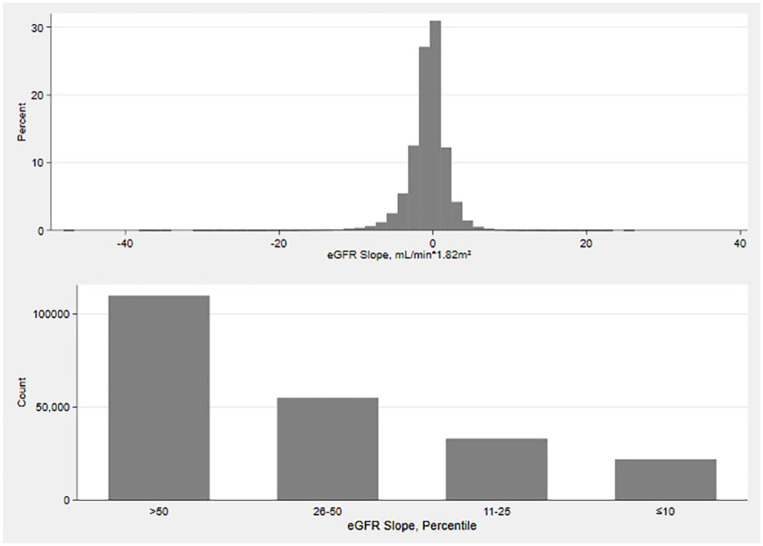
Distribution of the annual eGFR slope. eGFR estimated glomerular filtration rate. In the top figure, there is the distribution of eGFR slope (mL/min/1.73 m^2^ per year). In the bottom figure, each participant’s eGFR slope has been classified into 4 percentile bins: >50th percentiles, 26th-50th percentiles, 11th-25th percentiles, and ≤10th percentiles. Only the participants in the >50th percentiles have slopes that increase (as well as decrease). The other three bins only show declines in eGFR. The specific ranges are given in Table 1.

**Figure 4.**
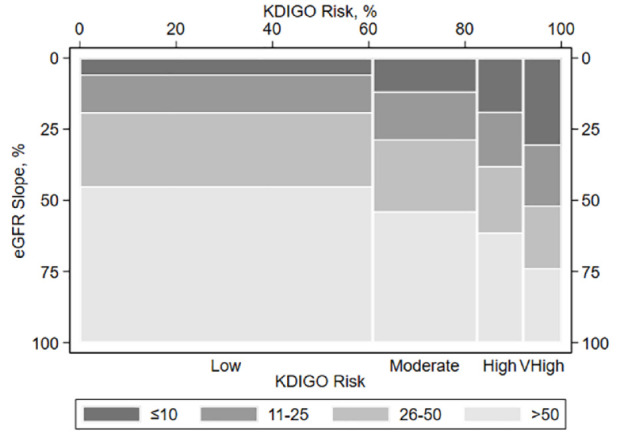
Joint distribution of KDIGO stage and the annual eGFR slope. CKD, chronic kidney disease; eGFR, estimated glomerular filtration rate; KDIGO, Kidney Disease Improving Global Outcomes. Each participant’s eGFR slope (mL/min/1.73 m^2^ per year) has been classified into 4 percentile bins: >50th percentiles (lightest gray shading), 26th-50th percentiles (medium-light gray shading), 11th-25th percentiles (medium-dark gray shading), and ≤10th percentiles (darkest gray shading). The largest eGFR declines are those in the ≤10th percentiles bin. The heights of the bars depict the percentage of participants who fall into these eGFR slope percentiles bins. The widths of the bars depict the percentage of participants with four levels of KDIGO risk: low, moderate, high, and very high.

The distribution of the annual eGFR slope was Gaussian (Figure 3); the median was −0.41 mL/min/1.73 m^2^ (IQR −1.67, 0.62). eGFR slope was missing for 34.8% of participants. In those with slopes, 61.0% had eGFR declines (i.e., eGFR slope <0 mL/min/1.73 m^2^). The spine plot demonstrates a larger proportion of participants with greater eGFR declines among those in greater KDIGO risk categories (Figure 4).

Medication dispensation by KDIGO risk and rate of the annual eGFR decline (Supplement Figure 2) demonstrated utilization of SGLT2-inhibitors in <25% of all participants. ACE inhibitor, ARB, and statin use was between 55% and 76%, with numerically greater use at moderate to very-high KDIGO risk compared with low KDIGO risk.

Participants at very-high KDIGO risk and with ≤10th percentiles of eGFR loss accounted for most participants that developed ESKD (Supplement Figure 3); they also contributed a disproportionately large amount to other outcomes. A similar pattern is observed for all-cause hospitalization and all-cause mortality (Supplement Figure 4).

In fully adjusted models, greater annual eGFR decline was significantly associated with several adverse clinical outcomes in participants with T2DM, independent of the components of KDIGO risk (baseline eGFR and albuminuria) and other covariates (Figure 2; Supplement Table 1). Several outcomes were significantly associated with eGFR declines in the 11th to the 25th percentiles (range −3.34, −1.67 mL/min/1.73 m^2^) compared to >50th percentiles (ESKD: OR 1.50, 95% CI 1.15, 1.96; and first stroke: OR 1.12, 95% CI 1.002, 1.26). When we compared participants with eGFR decline in the ≤10th percentiles (range ≤−3.34 mL/min/1.73 m^2^) to >50th percentiles, most clinical outcomes with the exception of first MI, hospitalization due to MI, and hospitalization due to stroke, were significantly associated with eGFR decline. When we modeled eGFR decline as a linear term, we found that every 1 mL/min/1.73 m^2^/ year decline at baseline was associated with most clinical outcomes in the order of 1% to 10% regardless of whether we used an indicator for missing slope or whether we assumed a slope of 0 for missing slopes. In sensitivity analyses, we considered participants with and without ACEi/ARB and/or SGLT2i prescriptions, and separately, we adjusted only for the covariates used in the KFRE^22^ (Supplement Table 2). The results for both analyses were very similar to the primary analysis.

The marginal risk of clinical outcomes by the annual eGFR slope (Table 2) in a fully adjusted model demonstrates the risk for adverse clinical outcomes by observed eGFR decline. Many outcomes, including end-stage kidney disease, have relatively modest increases in marginal risk; outcomes with larger increases in marginal risk with greater eGFR decline include heart failure, all-cause hospitalization, and cardiovascular and all-cause mortality.

**Table 2.** Marginal Risk of Clinical Outcomes by eGFR Slope.

Outcome	eGFR decrease, mL/min/1.73 m^2^/year					
	0	1	2	5	10	15
ESKD	0.2 (0.2, 0.2)	0.2 (0.2, 0.2)	0.2 (0.2, 0.3)	0.3 (0.3, 0.3)	0.4 (0.4, 0.4)	0.5 (0.4, 0.6)
New MI	0.4 (0.4, 0.4)	0.4 (0.4, 0.4)	0.4 (0.4, 0.4)	0.4 (0.3, 0.5)	0.4 (0.3, 0.6)	0.5 (0.2, 0.7)
New stroke	1.1 (1.0, 1.1)	1.1 (1.0, 1.1)	1.1 (1.1, 1.2)	1.2 (1.1, 1.3)	1.4 (1.2, 1.6)	1.6 (1.2, 2.0)
Heart failure	1.2 (1.2, 1.3)	1.3 (1.3, 1.3)	1.4 (1.4, 1.4)	1.7 (1.6, 1.9)	2.5 (2.2, 2.8)	3.6 (2.9, 4.3)
Hospitalization						
All-cause	12.3 (12.2, 12.4)	12.8 (12.7, 12.9)	13.3 (13.2, 13.5)	15.0 (14.7, 15.3)	18.2 (17.5, 19.0)	21.9 (20.6, 23.2)
Kidney	1.0 (0.9, 1.0)	1.0 (1.0, 1.0)	1.0 (1.0, 1.1)	1.2 (1.1, 1.3)	1.5 (1.3, 1.7)	1.9 (1.5, 2.3)
Cardiovascular	2.1 (2.0, 2.1)	2.1 (2.1, 2.2)	2.2 (2.2, 2.3)	2.5 (2.4, 2.6)	3.0 (2.7, 3.3)	3.6 (3.1, 4.1)
MI	0.4 (0.4, 0.5)	0.4 (0.4, 0.5)	0.4 (0.4, 0.5)	0.4 (0.4, 0.5)	0.5 (0.3, 0.6)	0.5 (0.3, 0.7)
Stroke	0.3 (0.3, 0.3)	0.3 (0.3, 0.3)	0.3 (0.3, 0.4)	0.4 (0.3, 0.4)	0.4 (0.3, 0.6)	0.5 (0.3, 0.7)
Heart failure	0.4 (0.4, 0.5)	0.5 (0.4, 0.5)	0.5 (0.5, 0.5)	0.6 (0.5, 0.6)	0.7 (0.6, 0.8)	0.9 (0.7, 1.1)
Mortality						
All-cause	2.1 (2.1, 2.2)	2.3 (2.2, 2.3)	2.5 (2.4, 2.5)	3.1 (3.0, 3.2)	4.4 (4.1, 4.8)	6.3 (5.6, 7.0)
Kidney	0.1 (0.0, 0.1)	0.1 (0.0, 0.1)	0.1 (0.0, 0.1)	0.1 (0.1, 0.1)	0.1 (0.1, 0.1)	0.1 (0.0, 0.2)
Cardiovascular	0.7 (0.6, 0.7)	0.7 (0.7, 0.7)	0.7 (0.7, 0.8)	0.9 (0.8, 1.0)	1.2 (1.0, 1.4)	1.6 (1.3, 2.0)

*Note.* ACE = angiotensin-converting enzyme; ARB = angiotensin II receptor blockers; eGFR = estimated glomerular filtration rate; ESKD = end-stage kidney disease; HbA1c = glycated hemoglobin; MI = myocardial infarction; SGLT2 = sodium-glucose cotransporter-2; TIA = transient ischemic attack.

Risk (%) with 95% confidence intervals are presented for Model 2 which parametrizes eGFR slopes that were negative with a linear term and an indicator for missing slope. Positive slopes were assigned a slope value of 0. The model also adjusted for baseline eGFR (≥90, 60-<90, 45-<60, 30-<45, 15-<30, <15 mL/min/1.73 m^2^, and missing), albuminuria (none/mild, moderate, severe, missing), glycated hemoglobin (<7%, 7-8%, >8-9%, >9%, and missing), age, biological sex, rural status, comorbidities (atrial fibrillation, heart failure, coronary artery disease, peripheral artery disease, stroke, and retinopathy) and prescriptions filled (SGLT2 inhibitors, ACE inhibitors, or ARBs, and statins).

## Discussion

In a population-based cohort of persons with T2DM, we determined the distribution of the annual slope of eGFR using a 3-year look back period, assessed the association of rate of decline with KDIGO risk categories, determined the association of KDIGO risk category and eGFR decline with clinically important outcomes, and assessed the independent association of eGFR decline at baseline on these outcomes with comprehensive adjustment including eGFR, albuminuria, comorbid conditions, A1c, and medication dispensation. A statistically significant association was observed between baseline rate of eGFR and clinically important adverse outcomes, including new diagnosis of heart failure, all-cause hospitalization, and cardiovascular and all-cause mortality. Our findings support the use of previous eGFR decline as measured by eGFR slope to assess risk not only of ESKD but also other clinically important adverse outcomes.

The median rate among the 61% of participants in whom the rate of the annual eGFR decline could be determined was relatively modest at −0.41 mL/min/1.73 m^2^; participants with eGFR slope in the 11th-25th and ≤10th percentile (median −2.31 and −4.71 mL/min/1.73 m^2^, respectively) comprised a larger proportion of subjects within the higher KDIGO risk categories. Those with a rate of eGFR decline at the ≤10th percentile (median −4.71 mL/min/1.73 m^2^) comprised approximately 5% and 10% of the low and moderate KDIGO risk categories, respectively, but comprised over 25% of the very-high-risk category. This may, in part, be due to the severity of albuminuria as it is used to define KDIGO risk and is associated with increased risk of clinical outcomes.

In fully adjusted models that account for albuminuria and other factors, annual rate of decline of eGFR remained statistically significant for most outcomes, indicating that rate of decline of eGFR alone may provide information on future risk. Rate of past eGFR decline in this patient population may be used by clinicians to inform future risk of kidney failure—this analysis supports this premise but also indicates that historical rate of eGFR decline is independently associated with new diagnosis of heart failure, hospitalization, and mortality, with 1% to 10% increase in the odds ratio for every 1 mL/min/1.73 m^2^ more rapid decline per year in eGFR. While CKD, defined by GFR and albuminuria, is well described as independently associated with cardiovascular outcomes, hospitalization, and mortality, rate of decline of eGFR may be yet another important characteristic of kidney disease that provides additional information on the risk of these events.

When the association of annual rate of eGFR decline is translated into marginal risk, it is notable that the absolute change in risk of adverse events varies by outcome. Compared with a rate of eGFR decline of −1 mL/min/1.73 m^2^ per year, in those with a decline in eGFR of −10 mL/min/1.73 m^2^ per year, the greatest absolute change in risk of events is noted for all-cause hospitalization (12.8 to 18.2), and all-cause mortality (2.3 to 4.4) and is relatively modest for ESKD (0.2 to 0.4; noting 1 year follow-up), which may be too short to fully characterize risk of kidney failure. While it might be anticipated that the additional information provided by consideration of rate of eGFR decline may be in risk of ESKD, our findings suggest that greater additional information is provided on risk of other outcomes; however, the absolute marginal risk for some outcomes may not be clinically significant.

Our findings are concordant with a recent study conducted in the United Kingdom among 30 222 subjects with T2DM in a UK primary health care setting. In adjusted analyses, eGFR slope of <−3 was associated with an increased risk of MACE, heart failure, and mortality.^23^ Previous studies in a general CKD population have described an association of decline in eGFR and outcomes including mortality and ESKD.^4-7^ This analysis confirms this association in a contemporary cohort, accounts for numerous factors, including treatment with ACEI/ARB and SLGT2i, considers additional clinically important outcomes, and further supports consideration of eGFR decline as a risk factor.

This study has several strengths, including a population-based cohort design, availability of laboratory data to define KDIGO risk and eGFR decline, and multiple approaches to examining the association of eGFR decline and outcome. Limitations include the use of administrative data, which may be influenced by measurement error and misclassification. Although validated approaches to exposure and outcome determination were used where possible, these algorithms do not have perfect sensitivity and specificity; for example, the algorithm for determining diabetes has a sensitivity of 80%. Further, data is missing in many participants; while the missingness of eGFR values to determine the rate of decline was explored in varying models, missingness of other lab findings including albuminuria may have influenced results. Further, slope of eGFR was calculated but did not explicitly characterize or incorporate episodes of AKI or non-linearity. In addition, while 1 year of follow-up may be sufficient for cardiovascular outcomes, it may be too short to fully characterize the risk of kidney failure. Finally, while this study was conducted in a large population-based setting, it was in one province and may not be generalizable to all jurisdictions.

## Conclusion

In this population-based cohort of participants with T2DM, rate of eGFR decline observed from the previous 3 years was associated with an increased risk of cardiovascular events including heart failure, hospitalization, and mortality (although causality cannot be assumed). While a greater proportion of participants at high or very-high KDIGO risk had greater eGFR rate of decline than those with less severe KDIGO risk, in fully adjusted analysis, eGFR rate of decline demonstrated a statistically significant association with adverse clinical outcomes. The clinical importance of eGFR rate of decline was greatest for outcomes of mortality, cardiovascular and all-cause hospitalization, and new diagnosis of heart failure. The absolute marginal risk may not be considered large for all of the outcomes considered. These findings support consideration of the rate of eGFR decline to inform risk stratification and may inform clinicians and policymakers to optimize treatment and inform health care system planning.

## Supplemental Material

sj-docx-1-cjk-10.1177_20543581241255781 – Supplemental material for Association of Glomerular Filtration Rate Decline With Clinical Outcomes in a Population With Type 2 Diabetes Supplemental material, sj-docx-1-cjk-10.1177_20543581241255781 for Association of Glomerular Filtration Rate Decline With Clinical Outcomes in a Population With Type 2 Diabetes by Scott W. Klarenbach, David Collister, Natasha Wiebe, Aminu Bello, Stephanie Thompson and Neesh Pannu in Canadian Journal of Kidney Health and Disease
