# Supplementary material for: Association of Glomerular Filtration Rate Decline With Clinical Outcomes in a Population With Type 2 Diabetes
Source: Can J Kidney Health Dis. 2024 Jun 10;11:20543581241255781. doi: 10.1177/20543581241255781 (PMC11163929; doi:10.1177/20543581241255781)
Supplement: sj-docx-1-cjk-10.1177_20543581241255781 – Supplemental material for Association of Glomerular Filtration Rate Decline With Clinical Outcomes in a Population With Type 2 Diabetes [file sj-docx-1-cjk-10.1177_20543581241255781.docx]

**Supplement Table 1.** **Adjusted odds ratios of clinical outcomes by eGFR slope**

| **Outcome** | **All** | **eGFR slope, percentiles (reference >50^th^ ^%ile^)** | | | **eGFR decrease, per 1 mL/min/1.73m^2^/year** | |
| --- | --- | --- | --- | --- | --- | --- |
|  | **N (%)** | **26-50^th^ ^%ile^** | **11-25^th^ ^%ile^** | **≤10^th^ ^%ile^** | **With missing**  **indicator** | **Without missing**  **indicator** |
| ESKD | 906 (0.3) | 1.13 (0.86,1.50) | 1.50 (1.15,1.96) | 2.22 (1.75,2.82) | 1.07 (1.06,1.09) | 1.07 (1.05,1.09) |
| New MI | 1,173 (0.4) | 0.92 (0.77,1.09) | 0.98 (0.80,1.20) | 0.98 (0.77,1.23) | 1.01 (0.98,1.05) | 1.00 (0.97,1.03) |
| New stroke | 3,222 (1.1) | 1.01 (0.91,1.12) | 1.12 (1.002,1.26) | 1.23 (1.08,1.40) | 1.03 (1.01,1.05) | 1.03 (1.01,1.05) |
| Heart failure | 3,999 (1.3) | 0.86 (0.78,0.95) | 1.08 (0.98,1.20) | 1.42 (1.27,1.59) | 1.08 (1.06,1.10) | 1.08 (1.06,1.10) |
|  |  |  |  |  |  |  |
| Hospitalization |  |  |  |  |  |  |
| All-cause | 43,142 (12.8) | 0.88 (0.86,0.91) | 0.99 (0.96,1.03) | 1.31 (1.26,1.36) | 1.05 (1.05,1.06) | 1.06 (1.05,1.07) |
| Kidney | 3,402 (1.0) | 0.90 (0.81,0.998) | 1.01 (0.90,1.13) | 1.34 (1.20,1.51) | 1.05 (1.03,1.06) | 1.05 (1.04,1.07) |
| Cardiovascular | 7,293 (2.2) | 0.98 (0.92,1.05) | 1.04 (0.96,1.13) | 1.28 (1.18,1.39) | 1.04 (1.03,1.05) | 1.04 (1.03,1.05) |
| MI | 1,448 (0.4) | 0.94 (0.81,1.10) | 0.97 (0.82,1.16) | 1.00 (0.82,1.22) | 1.01 (0.98,1.04) | 1.00 (0.97,1.03) |
| Stroke | 1,108 (0.3) | 1.08 (0.91,1.28) | 1.02 (0.83,1.24) | 1.18 (0.95,1.46) | 1.03 (0.999,1.06) | 1.03 (0.99,1.06) |
| Heart failure | 1,637 (0.5) | 0.90 (0.77,1.04) | 1.01 (0.87,1.19) | 1.32 (1.14,1.53) | 1.05 (1.03,1.07) | 1.05 (1.03,1.07) |
|  |  |  |  |  |  |  |
| Mortality |  |  |  |  |  |  |
| All-cause | 7,820 (2.3) | 0.79 (0.74,0.85) | 0.97 (0.89,1.05) | 1.56 (1.44,1.68) | 1.09 (1.08,1.10) | 1.10 (1.09,1.11) |
| Kidney | 191 (0.06) | 1.08 (0.68,1.72) | 0.99 (0.60,1.64) | 1.66 (1.06,2.60) | 1.05 (1.003,1.10) | 1.05 (0.996,1.10) |
| Cardiovascular | 2,431 (0.7) | 0.89 (0.78,1.01) | 1.10 (0.97,1.26) | 1.44 (1.26,1.64) | 1.07 (1.05,1.09) | 1.07 (1.05,1.09) |
|  |  |  |  |  |  |  |

ACE angiotensin-converting enzyme, ARB angiotensin II receptor blockers, eGFR estimated glomerular filtration rate, ESKD end-stage kidney disease, HbA1c glycated hemoglobin, MI myocardial infarction, SGLT2 sodium-glucose cotransporter-2, TIA transient ischemic attack.

Odds ratios with 95% confidence intervals are presented for various model parametrizations of eGFR slope. Model 1 parametrizes eGFR slope into 5 percentile bins: ≥50^th^ percentile (range -0.41 to 26.22 mL/min/1.73m^2^; referent), 26-50^th^ percentiles (range -1.6 to -0.41 mL/min/1.73m^2^), 11-25^th^ percentiles (range -3.34 to -1.67 mL/min/1.73m^2^), ≤10^th^ percentile (range -48.04 to -3.34 mL/min/1.73m^2^), and missing slope data (not shown). Model 2 parametrizes eGFR slopes that were negative with a linear term and an indicator for missing slope. Positive slopes were assigned a slope value of 0. Model 3 parametrizes eGFR slopes that were negative with a linear term. Positive and missing slopes were assigned a slope value of 0. All the models adjust for baseline eGFR (≥90, 60-<90, 45-<60, 30-<45, 15-<30, <15 mL/min/1.73m^2^, missing), albuminuria (none/mild, moderate, severe, missing), glycated hemoglobin (<7, 7-8, >8-9, >9%, missing), age, biological sex, rural status, comorbidities (atrial fibrillation, heart failure, coronary artery disease, peripheral artery disease, stroke, retinopathy) and prescriptions filled (SGLT2 inhibitors, ACE inhibitors or ARBs, statins).

**Supplement Table 2. Adjusted odds ratios of clinical outcomes by eGFR slope**

| **Outcome** | **eGFR decrease, per 1 mL/min/1.73m^2^/year with missing indicator** | | | |
| --- | --- | --- | --- | --- |
|  | **Primary** | **Sensitivity: no ACEi, ARB or SGLT2i users** | **Sensitivity: ACEi, ARB and/or SGLT2i users** | **Sensitivity: only KFRE covariates** |
| N | 336,376 | 140,436 | 195,940 | 336,376 |
| ESKD | 1.07 (1.06,1.09) | 1.07 (1.05,1.11) | 1.07 (1.05,1.09) | 1.07 (1.06,1.09) |
| New MI | 1.01 (0.98,1.05) | 1.00 (0.92,1.09) | 1.02 (0.98,1.06) | 1.02 (0.98,1.05) |
| New stroke | 1.03 (1.01,1.05) | 1.01 (0.97,1.05) | 1.04 (1.02,1.06) | 1.03 (1.01,1.05) |
| Heart failure | 1.08 (1.06,1.10) | 1.10 (1.07,1.13) | 1.07 (1.05,1.09) | 1.08 (1.06,1.10) |
|  |  |  |  |  |
| Hospitalization |  |  |  |  |
| All-cause | 1.05 (1.05,1.06) | 1.05 (1.04,1.06) | 1.06 (1.05,1.06) | 1.07 (1.06,1.07) |
| Kidney | 1.05 (1.03,1.06) | 1.04 (1.01,1.07) | 1.05 (1.03,1.07) | 1.05 (1.04,1.07) |
| Cardiovascular | 1.04 (1.03,1.05) | 1.03 (1.01,1.06) | 1.04 (1.03,1.06) | 1.06 (1.05,1.07) |
| MI | 1.01 (0.98,1.04) | 1.03 (0.96,1.10) | 1.00 (0.97,1.04) | 1.02 (0.995,1.05) |
| Stroke | 1.03 (0.999,1.06) | 0.95 (0.87,1.04) | 1.05 (1.01,1.08) | 1.04 (1.01,1.07) |
| Heart failure | 1.05 (1.03,1.07) | 1.05 (1.01,1.08) | 1.05 (1.03,1.07) | 1.09 (1.07,1.11) |
|  |  |  |  |  |
| Mortality |  |  |  |  |
| All-cause | 1.09 (1.08,1.10) | 1.10 (1.08,1.12) | 1.08 (1.07,1.10) | 1.11 (1.10,1.12) |
| Kidney | 1.05 (1.003,1.10) | 1.04 (0.97,1.11) | 1.08 (1.01,1.16) | 1.07 (1.03,1.12) |
| Cardiovascular | 1.07 (1.05,1.09) | 1.06 (1.03,1.09) | 1.07 (1.05,1.10) | 1.10 (1.08,1.12) |
|  |  |  |  |  |

ACE angiotensin-converting enzyme, ARB angiotensin II receptor blockers, eGFR estimated glomerular filtration rate, ESKD end-stage kidney disease, HbA1c glycated hemoglobin, KFRE Kidney Failure Risk Equation, MI myocardial infarction, SGLT2 sodium-glucose cotransporter-2, TIA transient ischemic attack.

Odds ratios with 95% confidence intervals are presented. The models parametrize eGFR slopes that were negative with a linear term and an indicator for missing slope. Positive slopes were assigned a slope value of 0. The primary model further adjusted for baseline eGFR (≥90, 60-<90, 45-<60, 30-<45, 15-<30, <15 mL/min/1.73m^2^, missing), albuminuria (none/mild, moderate, severe, missing), glycated hemoglobin (<7, 7-8, >8-9, >9%, missing), age, biological sex, rural status, comorbidities (atrial fibrillation, heart failure, coronary artery disease, peripheral artery disease, stroke, retinopathy) and prescriptions filled (SGLT2 inhibitors, ACE inhibitors or ARBs, statins). The first and second sensitivity models further adjusted for baseline eGFR (≥90, 60-<90, 45-<60, 30-<45, 15-<30, <15 mL/min/1.73m^2^, missing), albuminuria (none/mild, moderate, severe, missing), glycated hemoglobin (<7, 7-8, >8-9, >9%, missing), age, biological sex, rural status, comorbidities (atrial fibrillation, heart failure, coronary artery disease, peripheral artery disease, stroke, retinopathy) and statin prescription filled. Participants with SGLT2 inhibitor, ACE inhibitor or ARB use were not included. The third sensitivity model further adjusted for baseline eGFR (≥90, 60-<90, 45-<60, 30-<45, 15-<30, <15 mL/min/1.73m^2^, missing), albuminuria (none/mild, moderate, severe, missing), age, and biological sex – only the covariates used in the Kidney Failure Risk Equation.

**Supplement Figure 1. Study timeline**


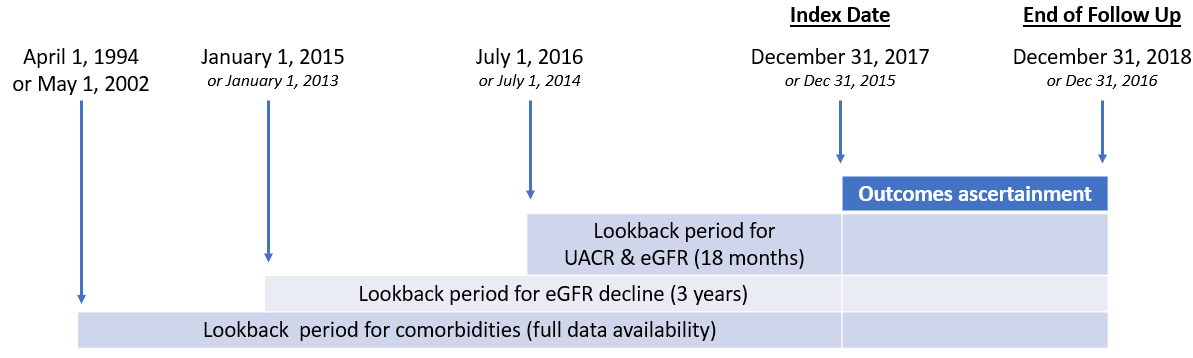


**Supplement Figure 2. Prescriptions by CKD stage and by eGFR slope**

**
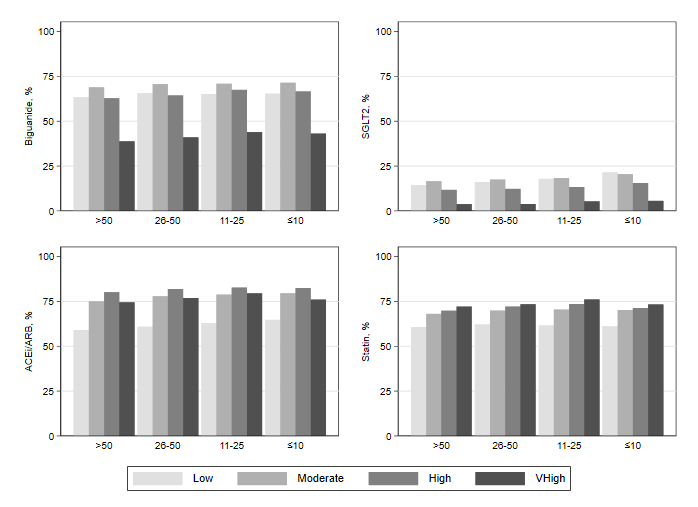
**

CKD chronic kidney disease, eGFR estimated glomerular filtration rate, ESKD end-stage kidney disease, HF heart failure, KDIGO Kidney Disease Improving Global Outcomes, MI myocardial infarction, TIA transient ischemic attack. The percentage of use and type of prescription is depicted on the y-axis. The x-axis shows the 4 percentile bins of eGFR slope (mL/min/1.73m^2^ per year) from left to right: >50^th^ percentiles, 26-50^th^ percentiles, 11-25^th^ percentiles, and ≤10^th^ percentiles. The largest eGFR declines are those in the ≤10^th^ percentiles bin. The shading depicts the number of participants that fall into the 4 KDIGO risk categories: low (lightest gray shading), medium (medium-light gray shading), high (medium-dark gray shading), and very high (darkest gray shading).

**Supplement Figure 3. Incident events (percentages) within each category of CKD stage and eGFR slope**

**
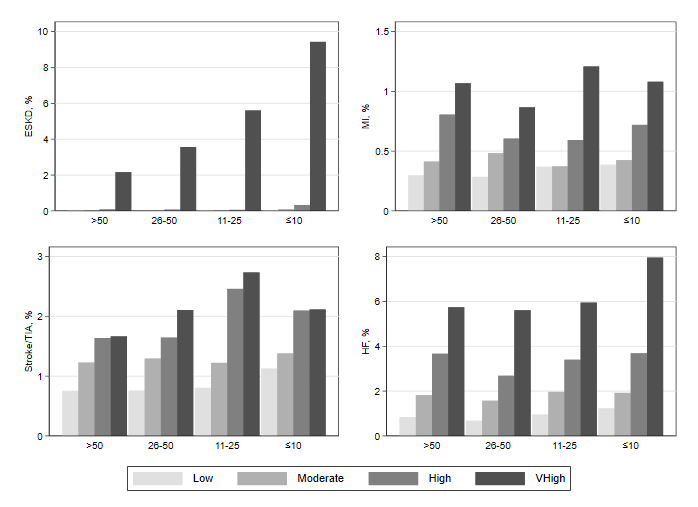
**

CKD chronic kidney disease, eGFR estimated glomerular filtration rate, ESKD end-stage kidney disease, HF heart failure, KDIGO Kidney Disease Improving Global Outcomes, MI myocardial infarction, TIA transient ischemic attack. The percentage and type of event is depicted on the y-axis. The x-axis shows the 4 percentile bins of eGFR slope (mL/min/1.73m^2^ per year) from left to right: >50^th^ percentiles, 26-50^th^ percentiles, 11-25^th^ percentiles, and ≤10^th^ percentiles. The largest eGFR declines are those in the ≤10^th^ percentiles bin. The shading depicts the number of participants that fall into the 4 KDIGO risk categories: low (lightest gray shading), medium (medium-light gray shading), high (medium-dark gray shading), and very high (darkest gray shading).

**Supplement Figure 4. Hospitalizations and mortality by CKD stage and eGFR slope**

**
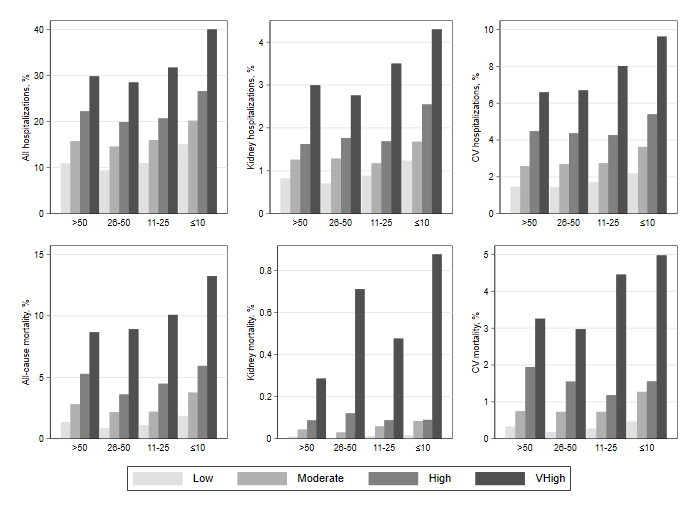
**

CKD chronic kidney disease, CV cardiovascular, eGFR estimated glomerular filtration rate, KDIGO Kidney Disease Improving Global Outcomes. The percentage and type of event is depicted on the y-axis. The x-axis shows the 4 percentile bins of eGFR slope (mL/min/1.73m^2^ per year) from left to right: >50^th^ percentiles, 26-50^th^ percentiles, 11-25^th^ percentiles, and ≤10^th^ percentiles. The largest eGFR declines are those in the ≤10^th^ percentiles bin. The shading depicts the number of participants that fall into the 4 KDIGO risk categories: low (lightest gray shading), medium (medium-light gray shading), high (medium-dark gray shading), and very high (darkest gray shading).
